# Management of diabetic ketoacidosis in children: Does early insulin glargine help improve outcomes?

**DOI:** 10.1111/1753-0407.13597

**Published:** 2024-08-13

**Authors:** Rebecca Ohman‐Hanson, G. Todd Alonso, Laura Pyle, Ryan McDonough, Mark Clements

**Affiliations:** ^1^ Pediatric Endocrinology University of Colorado School of Medicine, Anschutz Medical Campus and Children's Hospital Colorado Aurora Colorado USA; ^2^ Barbara Davis Center for Childhood Diabetes University of Colorado, Anschutz Medical Campus Aurora Colorado USA; ^3^ Department of Pediatrics University of Colorado School of Medicine Aurora Colorado USA; ^4^ Department of Biostatistics and Informatics Colorado School of Public Health Aurora Colorado USA; ^5^ Pediatric Endocrinology Children's Mercy Hospital Kansas City Missouri USA

**Keywords:** diabetic ketoacidosis, glargine, hypoglycemia, rebound hyperglycemia, recurrent ketosis

## Abstract

**Background:**

Rebound hyperglycemia following the resolution of diabetic ketoacidosis (DKA) is common in pediatric patients with type 1 diabetes, increasing the risk of recurrent DKA and complicating the transition to subcutaneous insulin. Multiple studies suggest that early administration of long‐acting insulin analogs during DKA management safely improves this transition.

**Objective:**

This study aimed to determine whether early insulin glargine administration in children with DKA prevents rebound hyperglycemia and recurrent ketosis without increasing the rate of hypoglycemia or hypokalemia.

**Methods:**

Patients aged <21 years presenting with DKA to Children's Mercy Kansas City between October 2012 and October 2016 were reviewed. They were categorized as Early (>4 h of overlap with intravenous [IV] insulin) and Late (<2 h of overlap) cohorts.

**Results:**

We reviewed 546 DKA admissions (365 Early and 181 Late). Rebound hyperglycemia (>180 mg/dL) was lower in the Early group (66% vs. 85%, *p* ≤ 0.0001). Hypoglycemia (<70 mg/dL) during IV insulin administration was higher in the Early group than in the Late group (27% vs. 19%, *p* = 0.042). Hypoglycemia within 12 h of IV insulin discontinuation was lower in the Early group (16% vs. 26%, *p* = 0.012). Recurrent ketosis, hypokalemia, and cerebral edema were not different between the groups.

**Conclusions:**

Early glargine administration in pediatric DKA management is safe, decreases the rate of rebound hyperglycemia, and improves the transition to subcutaneous insulin. Hypoglycemia is less frequent following IV insulin discontinuation with early glargine, but the IV insulin rate may need to be reduced to minimize hypoglycemia during IV insulin infusion.

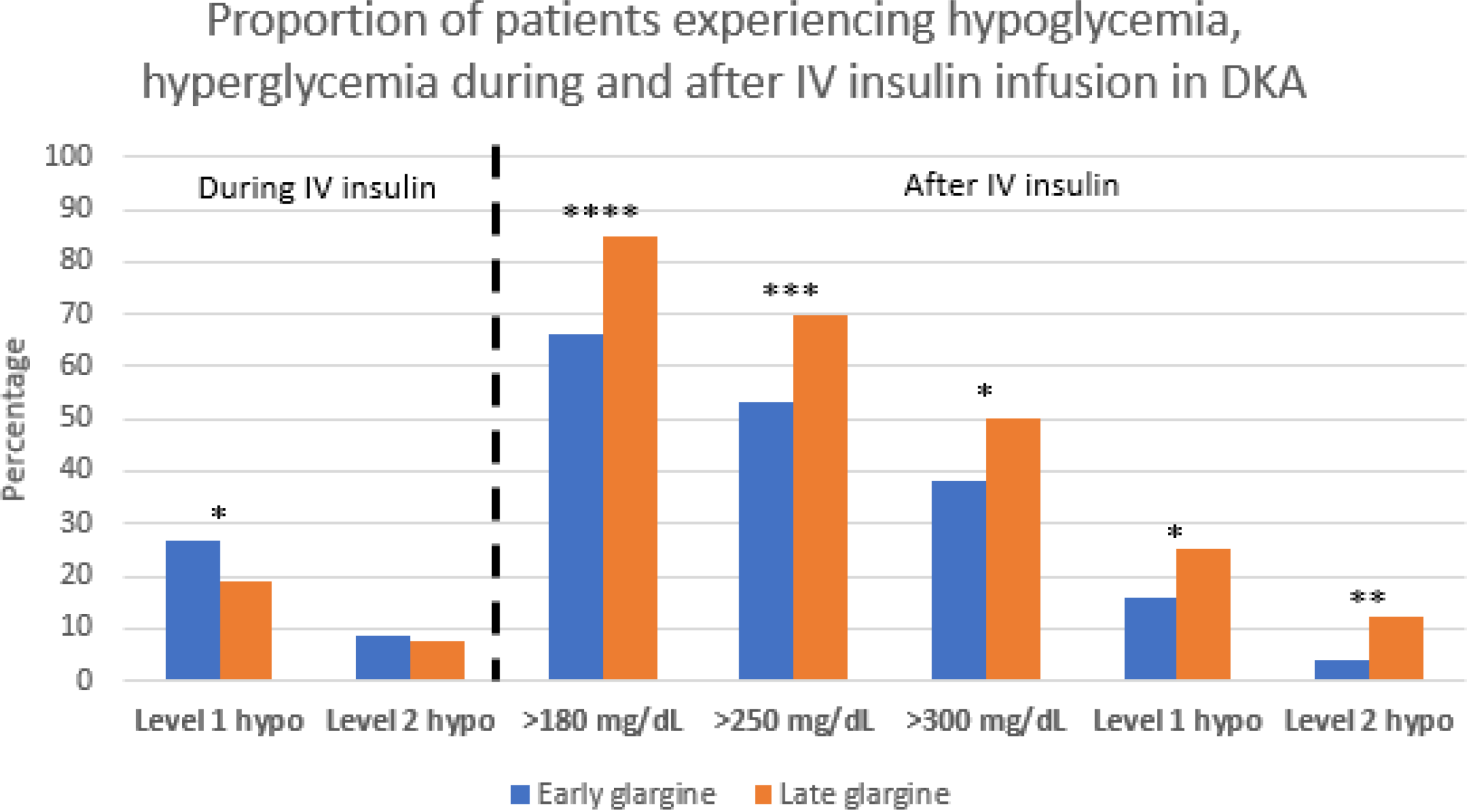

## Graphical abstract

Highlights

- Glargine insulin may be given early in diabetic ketoacidosis management, but pediatric data are sparse.
- Early administration of glargine insulin was associated with less rebound hyperglycemia but with more hypoglycemia during IV insulin infusion.

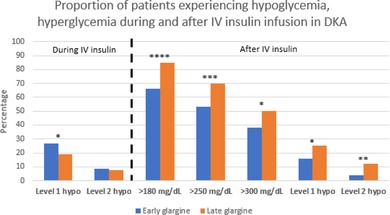

## 1 INTRODUCTION

Type 1 diabetes mellitus (T1D) is one of the most common chronic diseases in childhood, with an estimated prevalence of 1:400 by age 19 years and is increasing in children and adolescents by about 2.7% per year.^1^, ^2^ Diabetic ketoacidosis (DKA) remains the leading cause of morbidity and mortality in children with T1D.^3^ In the United States, the incidence of DKA in patients with established T1D is around 3%, whereas it ranges from 40% to 60% in those with newly diagnosed T1D.^4^, ^5^, ^6^, ^7^, ^8^ Treatment of DKA involves frequent laboratory monitoring as well as intravenous (IV) fluids and insulin. In the United States, it is responsible for over 500 000 hospital days for adult and pediatric patients, costing 2.4 billion USD annually.^9^, ^10^

A common complication of DKA associated with in‐hospital mortality and longer intensive care stay is rebound hyperglycemia, which occurs in 40%–90% of patients after a transition from IV to subcutaneous insulin^11^, ^12^, ^13^ and puts the patient at risk of recurrent ketosis and recurrence of DKA.^14^, ^15^

Insulin glargine, a long‐acting insulin analog, is peak‐less, takes 2 h to begin affecting blood sugar, and lasts for approximately 24 h.^16^ Owing to these properties, administering glargine several hours prior to the discontinuation of IV insulin may facilitate a smoother transition to subcutaneous insulin.^17^

The American Diabetes Association (ADA) and International Society of Pediatric and Adolescent Diabetes (ISPAD) provide slightly different advice on the timing of long‐acting insulin administration in relation to the end of IV insulin infusion, with the ADA recommending a 2‐h overlap and ISPAD suggesting an overlap is acceptable without specifying the timing.^18^, ^19^

Studies in adult patients suggest that glargine administration early in DKA management may improve the transition to subcutaneous insulin by accelerating the resolution of ketoacidosis, decreasing IV insulin duration and hospital length of stay, and reducing rebound hyperglycemia without increasing hypoglycemia.^12^, ^13^, ^20^, ^21^ Limited data in the pediatric population suggest that early glargine administration in DKA is feasible and results in a faster resolution of the acidosis and a lower total dose of IV insulin without an increased risk of hypoglycemia or cerebral edema,^22^, ^23^ though there may be an increased risk of hypokalemia.^23^ The effect of early glargine administration in children treated for DKA on rebound hyperglycemia or recurrent ketosis specifically is unknown. In this study, we aim to determine whether early glargine administration in children with DKA prevents rebound hyperglycemia and recurrent ketosis without increasing the rate of hypoglycemia or hypokalemia.

## 2 METHODS

Patients aged <21 years with new‐onset diabetes or preexisting type 1 diabetes presenting to Children's Mercy Kansas City (Kansas City, MO, USA) in DKA between October 2012 and October 2016 were eligible for inclusion in this retrospective chart review. This study was approved by the Institutional Review Board at Children's Mercy Kansas City and has been performed in accordance with the ethical standards laid down in the Declaration of Helsinki.

Early administration of glargine was defined as either (1) glargine administered before the start of IV insulin administration or (2) glargine administered after the start of IV insulin administration with >4 h of overlap with IV insulin. Late administration of glargine was administered with <2‐h overlap with IV insulin. These definitions were chosen to ensure adequate overlap in glargine action with the insulin infusion based on methods other authors have used.^23^ There were 142 patients whose glargine administration overlapped 2–4 h with IV insulin who were excluded from this analysis.

The primary outcome of this study was rebound hyperglycemia >180 mg/dL within 12 h of discontinuation of IV insulin. Secondary outcomes included rebound hyperglycemia >250 mg/dL and >300 mg/dL, median glucose within 12 h of IV insulin discontinuation, level 1 hypoglycemia (<70 mg/dL) either during IV insulin infusion or within 12 h of IV insulin discontinuation, level 2 hypoglycemia (<54 mg/dL) either during IV insulin infusion or within 12 h of IV insulin discontinuation, recurrent ketosis (blood beta‐hydroxybutyrate ≥1.5 mmol/L or moderate or large urine ketones within 12 h of IV insulin discontinuation), hypokalemia (serum potassium <3.5 mmol/L either during IV insulin administration or within 12 h of IV insulin discontinuation), and cerebral edema. Cerebral edema was defined clinically and extracted from the medical record.

Inclusion criteria were age <21 years at the time of clinical treatment, diagnosis of DKA (serum glucose or finger‐stick glucose concentration ≥200 mg/dL, serum bicarbonate concentration ≤15 mmol/L, and serum beta‐hydroxybutyrate ≥3.3 mmol/L), treated for DKA at the study site between October 2012 and October 2016, and diagnosis of type 1 diabetes mellitus. Type 1 diabetes was defined clinically as recorded in the medical record. At Children's Mercy Hospital, it is our practice to collect diabetes‐related autoantibodies on every patient diagnosed with diabetes.

Exclusion criteria included concurrent medications or metabolic dysfunction known to affect ketone production or glycemic response (type 2 diabetes mellitus, growth hormone deficiency, adrenal insufficiency, systemic steroids, and vasopressors).

From the medical record, we extracted age, weight, height, sex, race/ethnicity (self‐reported), admission and discharge times, new onset or preexisting diabetes diagnoses, insulin infusion start and end times, glargine dose and administration time, admission biochemical testing (serum glucose, HbA1c, serum potassium, serum beta‐hydroxybutyrate, and urine ketones), insurance coverage (private/military, public, and self‐pay), and median household income. Serial glucose and beta‐hydroxybutyrate measurements were collected for up to 12 h after insulin infusion discontinuation.

Management for DKA at Children's Mercy Kansas City is consistent with national and international guidelines.^14^, ^18^, ^19^ Once the diagnosis of DKA is made, treatment begins with IV fluid resuscitation followed by IV regular insulin at 0.1 units/kg/h. Basal insulin is given as soon as possible after the diagnosis of DKA. The dose is determined by whether the patient has a new diagnosis of diabetes or a prior history of diabetes. Patients with a new diagnosis of diabetes and those with preexisting diabetes who use an insulin pump receive 0.2–0.5 units/kg of glargine depending on age (0–4 years, 0.2 units/kg; 5–7 years, 0.3 units/kg; 8–10 years, 0.4 units/kg; 11 years and greater, 0.5 units/kg). Those who currently take long‐acting insulin injections are given their home dose. Serum glucose and ketone (beta‐hydroxybutyrate) levels are measured hourly, and serum potassium and bicarbonate are measured every 4 h. IV fluid concentrations are titrated as needed to maintain adequate serum potassium and glucose levels. Neurological assessment is performed hourly to monitor for cerebral edema. Insulin infusion is continued until bicarbonate is >17 mmol/L, the anion gap is normalized, and/or blood ketones are <0.6 mmol/L.

Biochemical tests were performed in the central chemistry laboratory at Children's Mercy Kansas City, including serum glucose, beta‐hydroxybutyrate, bicarbonate, potassium (Vitros), venous pH (Radiometer ABL), and HbA1c (HPLC).

Descriptive statistics include mean and standard deviations or percentiles for continuous variables, and frequencies and percentages for categorical variables. To compare characteristics in the Early and Late glargine groups, two‐tailed *t*‐tests or Mann–Whitney *U* tests were used for continuous variables, and the chi‐square test or Fisher's exact test was used for categorical variables. Statistical significance was defined as *p* < 0.05. Analyses were performed using the R software version 4.0 (R Core Team, Vienna).

## 3 RESULTS

Five hundred forty‐six episodes of DKA were included in the Early versus Late glargine analysis. There were 365 cases of DKA in the Early group and 181 in the Late group. In subanalyses separating the patients with and without newly diagnosed diabetes, there were 157 in the Early group and 18 in the Late group among patients with new‐onset diabetes and 208 versus 163, respectively, among those with established diabetes. Descriptive statistics are shown in Table 1. Overall, 58% were female (55% vs. 63% in the Early and Late groups, respectively; *p* = 0.095). Participants were 71% non‐Hispanic White, 15% African American, 4% Hispanic, and 10% reported as multiracial or other (*p* = 0.293). There were no significant differences between the groups regarding age, height, weight, median income, insurance carrier, glargine dose per kg, or HbA1c closest to admission. Sixty‐eight percent were newly diagnosed with onset type 1 diabetes. The mean glargine dose was 0.45 ± 0.14 units/kg and was not significantly different between the Early and Late groups (*p* = 0.395). The trend was similar when separating new‐onset and established cases.

**TABLE 1:** Descriptive statistics.

Characteristic	All (*n* = 546)	Early (*n* = 365)	Late (*n* = 181)	*p* value
Female	315 (58)	201 (55)	114 (63)	0.095
Ethnicity				
Non‐Hispanic White	390 (71)	270 (74)	120 (66)	0.293^a^
Non‐Hispanic Black	83 (15)	48 (13)	35 (19)	
Multiracial	39 (7)	24 (7)	15 (8)	
Hispanic	19 (4)	12 (3)	7 (4)	
Other	15 (3)	11 (3)	4 (2)	
Insurance				
Private/military	242 (44)	163 (45)	79 (44)	0.367^a^
Public	232 (42)	148 (41)	84 (46)	
Self‐pay	60 (11)	45 (12)	15 (8)	
Unknown	12 (2)	9 (2)	3 (2)	
New onset	371 (68)	208 (57)	163 (90)	<0.0001
Age	12.1 ± 4.5	11.9 ± 4.6	12.3 ± 4.3	0.412
Height, cm	146.5 ± 27.9	146.2 ± 29.5	146.9 ± 24.5	0.792
Weight, kg	45.6 ± 21.9	45.6 ± 22.5	45.5 ± 20.7	0.977
HbA1c, %	12.4 ± 2.2	12.4 ± 2.3	12.4 ± 2.0	0.971
Median income ($)	57 597 ± 28 599	57 552 ± 27 350	57 688.28 ± 31 046	0.960
Glargine (units/kg)	0.45 ± 0.14	0.45 ± 0.15	0.46 ± 0.14	0.395

*Note*: Statistics are *N* (%), mean ± SD, or median (IQR).

Abbreviation: IQR, Interquartile range.

^a^ Fisher's exact test.

**FIGURE 1:**
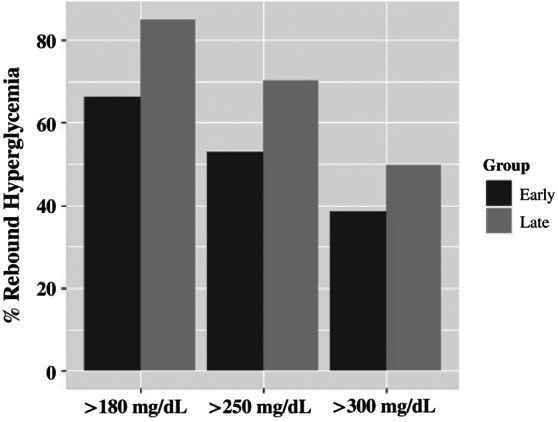
Percent of rebound hyperglycemia within 12 h of IV insulin discontinuation in Early versus Late glargine administration. Serum glucose cutoffs of >180, >250, and >300 mg/dL. *p* values for Early versus Late: >180 mg/dL, *p* < 0.0001; >250 mg/dL, *p* = 0.0002; >300 mg/dL, *p* = 0.015. IV, intravenous.

Rebound hyperglycemia >180 mg/dL was observed in 73% of all DKA admissions and was lower in the Early group (66% vs. 85%, *p* < 0.0001 [Figure 1]). Rebound hyperglycemia >250 mg/dL and >300 mg/dL were also lower in the Early group (53% Early vs. 70% Late; *p* = 0.0002, and 38% vs. 50%; *p* = 0.015, respectively [Figure 1]). These remained significant among patients with established diabetes and trended in the same direction despite *p* > 0.05 among those with newly diagnosed diabetes. There was no difference in median glucose within 12 h after IV insulin (*p* = 0.643), though it was lower (173 mg/dL in the Early group vs. 194 mg/dL in the Late group, *p* = 0.01) among those with established diabetes. Median glucose was slightly higher among those with new‐onset diabetes, 215 mg/dL, though not different between the Early and Late groups.

**FIGURE 2:**
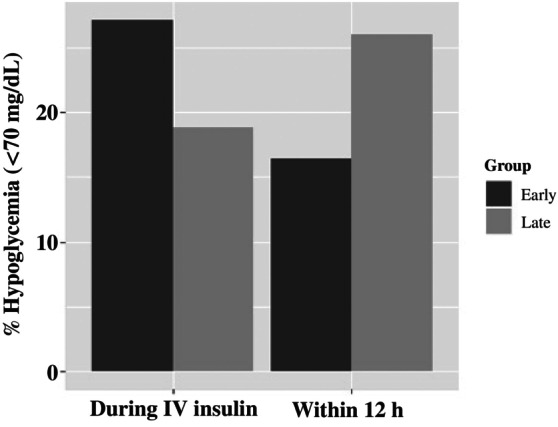
Percent of DKA admissions with level 1 hypoglycemia <70 mg/dL during IV insulin administration or within 12 h of IV insulin discontinuation in Early versus Late glargine administration. *p* values: during IV insulin, *p* = 0.042; within 12 h of IV insulin discontinuation, *p* = 0.012. DKA, diabetic ketoacidosis; IV, intravenous.

Level 1 hypoglycemia during IV insulin administration was observed in 24% of all DKA admissions and was higher in the Early group (27% vs. 19%; *p* = 0.042) (Figure 2). It was significantly different (*p* = 0.011) among those with established diabetes but not among those with newly diagnosed diabetes. Level 2 hypoglycemia during IV insulin administration was not different between the study groups (*p* = 0.806).

**FIGURE 3:**
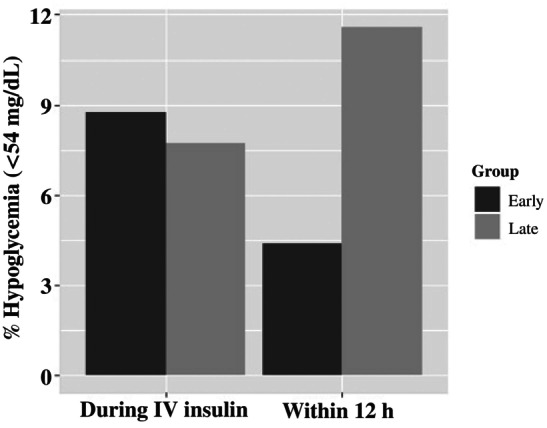
Percent of DKA admissions with level 2 hypoglycemia (serum glucose <54 mg/dL) during IV insulin administration or within 12 h of IV insulin discontinuation in Early versus Late glargine administration. *p* values: during IV insulin, *p* = 0.806; within 12 h of IV insulin discontinuation, *p* = 0.003. DKA, diabetic ketoacidosis; IV, intravenous.

Within 12 h of IV insulin discontinuation, level 1 hypoglycemia was observed in 20% of all DKA admissions and was lower in the Early group (16% vs. 26%; *p* = 0.012) (Figure 2). Level 2 hypoglycemia within 12 h of IV insulin discontinuation was also lower in the Early group (4% vs. 12%; *p* = 0.003) (Figure 3). Neither of these findings was significantly different in the subanalyses of new or established patients.

**FIGURE 4:**
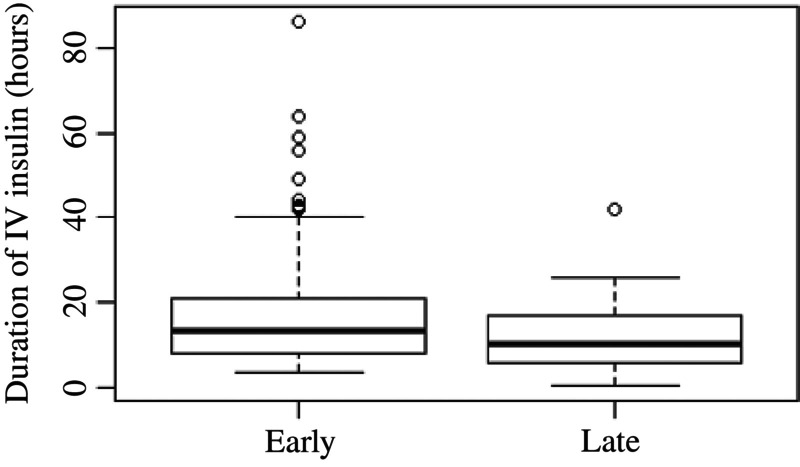
Mean IV insulin duration (h) between Early versus Late glargine administration (15.8 ± 10.3 vs. 11.4 ± 7.2; *p* < 0.0001). IV, intravenous.

Recurrent ketosis within 12 h of IV insulin discontinuation was observed in 44% of all DKA admissions but was not statistically different between the groups (39% Early vs. 49% Late; *p* = 0.121). However, it was significantly different (26.9% vs. 45%, *p* = 0.011) among those with established diabetes. Cerebral edema was diagnosed in eight patients, four of whom had established diabetes, without a difference between the Early and Late groups (*p* = 0.908). Hypokalemia was also not different between the groups either during IV insulin (23%, *p* = 0.242) or within 12 h of IV insulin discontinuation (31%, *p* = 0.356). The glucose decrease rate on IV insulin was faster in the Late group (median, 0.24 mg/dL [interquartile range {IQR}, 0.10–0.46] per minute vs. 0.14 mg/dL [0.06–0.31] per minute; *p* = 0.001) but was nonsignificant in the newly diagnosed and established diabetes subanalyses. Mean IV insulin duration was 15.8 ± 10.3 h versus 11.4 ± 7.2 h in the Early versus Late groups (*p* < 0.0001) (Figure 4) and was significantly different among established patients (12.2 vs. 11.3 h, respectively, *p* < 0.001) but not among those with newly diagnosed diabetes, though the trend was similar (16.7 vs. 12.6 h, respectively, *p* = 0.153).

## 4 DISCUSSION

This is the largest pediatric study to date of patients treated with glargine early in DKA management and the only pediatric study that evaluated glycemia and ketosis for up to 12 h following IV insulin discontinuation. Rebound hyperglycemia as DKA resolves is common in the pediatric population and is reduced by 19% with early administration of insulin glargine. This is consistent with studies in adults.^12^ Additionally, this pattern is preserved when assessing moderate (>250 mg/dL) and severe (>300 mg/dL) rebound hyperglycemia, which is more clinically relevant to the pediatric population. Recurrent ketosis was also common but not statistically significant between the Early and Late glargine groups. Therefore, a dose of glargine early in DKA management may help prevent the recurrence of DKA in children with type 1 diabetes.

Level 1 hypoglycemia (glucose <70 mg/dL) was identified more often in the Early glargine group during IV insulin infusion, but this trend reversed for both level 1 and level 2 hypoglycemia in the 12 h following IV insulin discontinuation. Other adult and pediatric studies have shown no difference in hypoglycemia,^12^, ^13^, ^20^, ^21^, ^23^ although one did observe a lower but nonstatistically different minimum glucose concentration during IV insulin in those receiving early glargine.^23^ Importantly, we found no difference in rates of level 2 hypoglycemia (glucose <54 mg/dL) between the Early and Late groups while on IV insulin.

The IV insulin infusion rate suggested by the hospital's clinical guidelines was 0.1 units/kg per hour when blood sugar was ≥80 mg/dL; however, 0.05 units/kg per hour is noninferior^24^, ^25^, ^26^ and is now included as acceptable in current ADA and ISPAD guidelines for the treatment of DKA in children.^14^, ^19^, ^27^

Continuing the IV insulin infusion without reducing its rate when glargine administration overlaps by several hours means the child is exposed to greater insulin action, which may increase the risk for hypoglycemia while on IV insulin, and it is consistent with our observation. Therefore, clinicians who choose to administer glargine early during DKA management should continue to monitor blood sugar values closely and optimize dextrose infusion, then consider decreasing the rate of IV insulin infusion if the glucose levels continue to fall too quickly. The fact that level 1 and level 2 hypoglycemia occurred less often in the 12 h following IV insulin discontinuation was reassuring and may encourage practice change to early glargine administration. We were not able to obtain reliable data for rapid‐acting insulin doses to be able to assess whether there was an association between bolus doses of rapid‐acting insulin for hyperglycemia and subsequent hypoglycemia, but this is a plausible mechanism. These questions should be further investigated with a prospective study.

Counter to other pediatric studies, we found no increased risk of hypokalemia in the Early glargine group.^13^, ^23^ A potential reason for this could be slight differences in the potassium content of the IV replacement fluids between centers or could be related to differences in the initial potassium concentration of patients at presentation between centers. Children's Mercy Kansas City used 20 mEq/L of potassium acetate and 20 mEq/L of potassium phosphate in their fluids. Reassuringly, no clinical complications of hypokalemia were noted in either of these prior studies.

The shorter duration of IV insulin in the Late glargine group was unexpected. We had anticipated that the timing of IV insulin suspension would be more flexible and, therefore, potentially earlier in many instances where glargine had already been administered. Indeed, the potential to simplify care by allowing earlier and more flexible IV insulin suspension has been suggested by our clinicians as a reason to advocate for early glargine administration. We suspect that selection bias contributed, and a randomized study could clarify this finding.

The faster rate of glucose decrease in the Late glargine group appears to be linked to the fact that we evaluated glucose decrease over the entire duration of IV insulin use, which was shorter in the Late glargine group. A glucose plateauing near the end of the IV duration may have been longer in the Early glargine group, which saw longer IV insulin duration. Data were collected from a single study site (Kansas City, MO) and among patients in a limited age range (7–16 years) and therefore may not be generalizable to the greater population.

## 5 CONCLUSIONS

Early glargine administration in pediatric DKA management is feasible and well‐tolerated, and it decreases rebound hyperglycemia, which could reduce hospital length of stay and DKA‐related morbidity. However, there may be an increased risk of level 1 hypoglycemia during IV insulin administration in patients whose glargine administration overlaps with IV insulin administration. Therefore, providers could consider using a lower IV insulin rate (such as 0.05 units/kg/h) as glucose levels fall, to avoid hypoglycemia. There does not appear to be a greater risk of level 2 hypoglycemia, and this risk does not continue after discontinuation of the IV insulin. Additional studies in this population, including randomized‐controlled trials, are needed.

## CONFLICT OF INTEREST STATEMENT

R.O.‐H., G.T.A., L.P., and R.M. have no disclosures to report. M.C. reports consulting feeds from Glooko, Inc and research support from Dexcom and Abbott Diabetes Care.
